# DIA Quantitative Proteomic Analysis Reveals Early Proteomic Responses to Honeybee-Derived Melittin in Murine Breast Cancer Cells

**DOI:** 10.3390/biology15191727

**Published:** 2026-10-01

**Authors:** Junling Zhu, Wenhua Xu, He Zang, Yunzhen Yang, Nian Fan, Yuwei Zhang, Yaqin Gao, Jianfeng Qiu, Dafu Chen, Rui Guo

**Affiliations:** 1 Ningbo No. 2 Hospital, Wenzhou Medical University, Ningbo 315000, China; zhujunling09@163.com; 2 National & Local United Engineering Laboratory of Natural Biotoxin, Fuzhou 350002, China; xwenhua0911@163.com (W.X.); zanghe321@163.com (H.Z.); yangyunzhen0120@163.com (Y.Y.); 13595105054@163.com (N.F.); 13759839701@163.com (Y.Z.); 18789833015@163.com (Y.G.); jfqiu@fafu.edu.cn (J.Q.); 3 Apitherapy Research Institute, Fujian Agriculture and Forestry University, Fuzhou 350002, China; 4 College of Bee Science, Fujian Agriculture and Forestry University, Fuzhou 350002, China

**Keywords:** melittin, breast cancer, honeybee venom, E0771 cells, quantitative proteomics, differentially abundant proteins

## Abstract

Breast cancer remains a major clinical challenge because of its molecular heterogeneity, therapeutic resistance, and disease recurrence. Melittin, the principal bioactive peptide of honeybee venom, exhibits antitumor activity in multiple experimental models; however, its early proteome-wide effects in breast cancer cells remain incompletely characterized. In this study, E0771 murine mammary carcinoma cells were exposed to melittin for 15 min. Colony formation and CCK-8 assays using 0–8 μg/mL melittin showed concentration-dependent reductions in clonogenic capacity and cell viability. For DIA-based quantitative proteomic profiling, E0771 cells were treated with 4 μg/mL melittin for 15 min. Among 9748 quantified protein entries, 339 proteins met the exploratory criteria for candidate differential abundance, including 114 up-regulated and 225 down-regulated candidates. GO and KEGG analyses associated these candidate proteins with cell junctions, cytoskeletal organization, protein kinase-related functions, metabolic processes, and cell-death-associated pathways. A STRING-derived association network containing 132 nodes and 216 edges further prioritized CASP8, RPS27A, ANXA5, and PIK3CA as relatively high-connectivity candidates. Collectively, these findings describe early proteomic responses to short-term melittin exposure and provide candidate proteins and biological processes for subsequent mechanistic validation rather than establishing causal molecular mechanisms.

## Simple Summary

Breast cancer remains one of the most common cancers worldwide, and the development of new therapeutic approaches is still urgently needed. Melittin, a natural peptide derived from honeybee venom, has attracted attention because of its potential ability to inhibit cancer cells. However, the overall changes occurring inside breast cancer cells after melittin treatment are not fully understood. Here, we investigated the effects of melittin on a mouse breast cancer cell model and explored the alterations in cellular proteins after short-term exposure. We found that melittin reduced the ability of breast cancer cells to grow and survive. By analyzing thousands of cellular proteins, we identified broad changes associated with cell structure, communication, metabolism, and processes related to cell death. Several important protein candidates were further identified and their changes were confirmed using an independent experimental method. These findings provide a comprehensive view of how melittin affects breast cancer cells and offer valuable information for future studies exploring honeybee venom-derived compounds as potential anticancer agents.

## 1. Introduction

Breast cancer is a highly heterogeneous malignancy that remains a leading cause of cancer-related morbidity and mortality among women worldwide. Among its molecular subtypes, triple-negative breast cancer (TNBC) represents the most aggressive form, largely due to the absence of effective targeted therapies [1]. Murine mammary carcinoma models provide valuable platforms for investigating tumor-cell responses to candidate anticancer agents under controlled experimental conditions. Among these models, E0771 cells are widely used for studying breast cancer biology, tumor growth, and therapeutic responses [2]. E0771 cells were originally derived from a spontaneous mammary tumor in C57BL/6 mice and have been extensively applied in syngeneic breast cancer studies; however, their molecular classification remains under discussion since different studies have reported variable receptor profiles and phenotypic characteristics. Therefore, the present study does not assign E0771 cells to a specific human breast cancer subtype but uses this model to investigate early cellular responses to melittin exposure. Current treatment strategies, including chemotherapy, endocrine therapy, and immunotherapy, often suffer from limited efficacy, systemic toxicity, and the emergence of drug resistance. These limitations underscore the urgent need for novel therapeutic agents and alternative treatment strategies [1,3].

Melittin, the major cytolytic peptide of honeybee venom, has emerged as a promising anticancer candidate and has demonstrated antitumor activity in a range of preclinical cancer models [4,5]. Recent reviews have further summarized the multifaceted anticancer activities of melittin, including membrane disruption, modulation of intracellular signaling networks, oxidative stress regulation, and induction of cell death-associated responses, highlighting its potential as a bioactive peptide for cancer therapy [6]. Previous studies have demonstrated that melittin can disrupt cancer cell membrane integrity and influence apoptosis-associated signaling and oxidative stress responses. However, whether these mechanisms represent universal responses across various cancer models or how they are coordinated at the global protein-network level remain unclear [7,8,9]. To overcome its systemic toxicity and improve therapeutic efficacy, various nanoparticle-based delivery systems have been developed, which can not only reduce off-target effects but also enhance its immunotherapeutic activity. In breast cancer, melittin has shown particular promise against TNBC, where it can suppress tumor cell proliferation as well as migration and sensitize cells to conventional therapies. These effects are believed to involve the modulation of signaling pathways related to lipid metabolism and cell survival [4,10,11]. However, despite encouraging preclinical findings, the molecular mechanisms underlying melittin’s anticancer activity in breast cancer remain poorly understood. Previous studies have primarily focused on its general cytotoxic effects, with limited exploration of its molecular targets and the downstream signaling events in breast cancer cells [12,13].

Proteomic approaches have become indispensable tools in cancer research, offering comprehensive insights into tumor biology, biomarker identification, and therapeutic target discovery. Recent advances in cancer proteomics have further demonstrated that global protein-expression landscapes can reveal clinically relevant molecular features, signaling alterations, and potential therapeutic vulnerabilities in breast cancer [14]. Large-scale proteomic profiling has revealed distinct molecular subtypes of breast cancer, linking protein expression patterns to tumor progression, metastasis, and treatment response [13,15,16]. For instance, dysregulated phosphorylation events and aberrant kinase activation have been implicated in tumorigenesis and therapy resistance [17]. Despite these advances, proteomic insights into the effects of melittin on breast cancer cells remain scarce. The global alterations in protein expression and the regulatory mechanisms induced by melittin at the cellular level are still largely undefined [12,18]. However, previous studies of melittin-mediated anticancer activity have mainly focused on individual signaling molecules or selected pathways, such as apoptosis-related regulators, growth-factor receptor signaling, or metabolic alterations. Consequently, the coordinated protein-abundance changes occurring immediately after melittin exposure remain poorly characterized [12,13,18]. Early proteomic responses may offer important insights into the initial cellular events triggered by melittin before secondary consequences relevant to prolonged cytotoxicity become dominant.

Compared with late-stage analyses performed after prolonged drug exposure, short-term proteomic profiling allows investigation of immediate molecular remodeling events, including changes in structural organization, signaling regulation, metabolic adaptation, and stress-associated responses.

In this study, the effects of melittin on the clonogenic capacity and viability of E0771 cells were first evaluated using colony formation and CCK-8 assays. CCK-8 assay was used to quantitatively evaluate cellular metabolic activity and viability after melittin exposure, whereas DIA-based quantitative proteomics was applied to comprehensively characterize global protein-abundance changes associated with early cellular responses. DIA-based quantitative proteomics was subsequently performed to characterize early protein abundance changes after a 15 min melittin exposure. We hypothesized that short-term melittin exposure induces coordinated alterations in protein networks associated with cellular structure, signaling regulation, metabolism, and cell-death-related processes before the establishment of prolonged cytotoxic effects. Hence, this study combined phenotypic assays with DIA-based quantitative proteomics to characterize early protein-abundance changes induced by melittin in E0771 cells. Our data not only provide a valuable proteomics resource for understanding melittin-mediated regulation in breast cancer cells, but also facilitate further mechanistic investigation and development of melittin-based therapeutic strategies.

## 2. Materials and Methods

### 2.1. Experimental Materials

The murine mammary carcinoma cell line E0771 was obtained from Procell Life Science & Technology Co., Ltd. (Wuhan, China) and authenticated by short tandem repeat (STR) profiling to confirm its identity and rule out cross-contamination. Dulbecco’s Modified Eagle Medium (DMEM, high glucose), fetal bovine serum (FBS), and 0.25% trypsin–EDTA were purchased from Gibco (Thermo Fisher Scientific, Waltham, MA, USA). PBS and penicillin–streptomycin were purchased from YEASEN Biotechnology (Shanghai, China). Cell Counting Kit-8 is from Beyotime (Shanghai, China). Melittin was purchased from Selleck (Houston, TX, USA). RIPA lysis buffer, protease inhibitor cocktail, and phosphatase inhibitors A and B were supplied by Epizyme Biotech (Shanghai, China). The 4% paraformaldehyde fixative, crystal violet staining solution from Beyotime Biotechnology (Shanghai, China).

### 2.2. Cell Culture

E0771 cells were maintained in DMEM supplemented with 10% (*v*/*v*) FBS and 1% (*v*/*v*) penicillin–streptomycin at 37 °C in a humidified incubator with 5% CO_2_. When cultures reached approximately 80% confluence, cells were rinsed twice with PBS and digested with 0.25% trypsin–EDTA at 37 °C. The reaction was terminated with complete medium containing FBS once cells became rounded and detached. Single-cell suspensions were prepared by gentle pipetting and subcultured at a 1:3 ratio. Cells in the logarithmic growth phase with >90% viability were used for subsequent experiments.

### 2.3. Colony Formation Assay

The long-term proliferative capacity of E0771 cells following short-term melittin exposure was evaluated using a colony formation assay. Cells were seeded into six-well plates at a density of 500 cells per well and allowed to adhere overnight. The cells were subsequently treated with melittin at concentrations of 0, 1, 2, 4, or 8 μg/mL for 15 min. After treatment, the melittin-containing medium was removed, and the cells were gently washed twice with phosphate-buffered saline. Fresh complete growth medium without melittin was then added, and the cells were cultured for 7 days to allow colony formation. The culture medium was replaced every 2–3 days.

At the end of the incubation period, the colonies were washed twice with phosphate-buffered saline, fixed with 4% paraformaldehyde for 15 min at room temperature, and stained with crystal violet staining solution for 20 min. Excess dye was removed by washing with PBS, and the plates were air-dried. Colonies containing at least 50 cells were counted. Colony formation efficiency was calculated as the number of colonies divided by the number of seeded cells and expressed as a percentage.

### 2.4. Cell Viability Assay

E0771 cells were seeded into 96-well plates at a density of 2 × 104 cells per well and allowed to adhere overnight. Cells were treated with 0, 1, 2, 4, or 8 μg/mL melittin for 15 min. Subsequently, 100 μL of CCK-8 reagent (Cell Counting Kit-8, Beyotime, Shanghai, China; C0038) was added to each well, and the plates were incubated for 30 min at 37 °C. Absorbance was measured at 450 nm using a microplate reader (Agilent Technologies, Santa Clara, CA, USA). Cell viability was normalized to that of the untreated control group, which was defined as 100%. Each treatment included 3 technical replicates, and the experiment was independently repeated three times. Statistical significance was evaluated using one-way ANOVA followed by Tukey’s multiple-comparison test. Normality and homogeneity of variance were assessed before parametric analysis.

### 2.5. Cell Treatment and Protein Extraction

Melittin was freshly dissolved in DMEM and diluted to a final concentration of 4 μg/mL immediately before use. E0771 cells in the logarithmic growth phase were seeded into six-well plates at a density of 5 × 10^5^ cells per well and cultured for 24 h at 37 °C in a humidified atmosphere containing 5% CO_2_. When the cells reached approximately 70–80% confluence, they were assigned to an untreated control group and a melittin-treated group, with three independent biological replicates per group. Cells in the melittin-treated group were exposed to 1 mL of DMEM containing 4 μg/mL melittin for 15 min, whereas control cells received an equal volume of DMEM without melittin under otherwise identical conditions.

After treatment, the medium was removed, and the cells were washed twice with ice-cold PBS. The cells were collected using a prechilled cell scraper in 1 mL PBS and transferred into 1.5 mL microcentrifuge tubes maintained on ice. Following centrifugation at 14,000 rpm for 5 min at 4 °C, the supernatant was discarded, and the cell pellets were lysed in 200 μL RIPA buffer supplemented with 1% protease inhibitor cocktail, 2 μL phosphatase inhibitor A, and 2 μL phosphatase inhibitor B. The samples were vortexed for 10 s, incubated on ice for 30 min, and subjected to ultrasonic disruption in an ice–water bath at 200 W using 10 cycles of 3 s on and 5 s off. The lysates were subsequently centrifuged at 14,000 rpm for 5 min at 4 °C, and the supernatants containing total cellular proteins were collected and stored at −80 °C until further analysis.

Three independent biological replicates were included in each proteomic group. Each replicate was independently cultured, treated, harvested, and processed for proteomic analysis. The sample size was selected for an exploratory discovery-phase proteomic study rather than for confirmatory hypothesis testing.

The relatively small number of biological replicates (*n* = 3 per group) limits statistical power, particularly after correction for multiple testing, and may increase the uncertainty of individual candidate protein associations.

### 2.6. Protein Quality Assessment and Peptide Preparation

Protein concentrations were determined using the Bradford assay. Bovine serum albumin standards and appropriately diluted protein samples were mixed with Coomassie Brilliant Blue G-250 reagent in 96-well plates, and absorbance was measured at 595 nm using a Synergy H1 multimode microplate reader (Agilent Technologies, Santa Clara, CA, USA). Protein concentrations were calculated from the corresponding standard curve.

Protein integrity and sample consistency were evaluated by 4–12% SDS–PAGE. Briefly, 10 μg of protein from each sample was mixed with loading buffer, heated at 95 °C for 5 min, and centrifuged at 20,000× *g* for 5 min. Proteins were separated at 80 V for 20 min, followed by 120 V for 60 min. The gels were stained with Coomassie Brilliant Blue and imaged after destaining.

For enzymatic digestion, 150 μg of protein from each sample was reduced with 10 mM dithiothreitol and alkylated with 20 mM iodoacetamide. The proteins were subsequently digested with sequencing-grade trypsin (V5280; Promega, Madison, WI, USA) at a protein-to-enzyme ratio of 50:1 (*w*/*w*) for 14–16 h at 37 °C. The resulting peptides were desalted using solid-phase extraction cartridges (Waters Corporation, Milford, MA, USA), vacuum-dried, reconstituted in ultrapure water, and stored at −20 °C until LC–MS/MS analysis.

### 2.7. DIA-LC–MS/MS Analysis and Data Processing

Dried peptide samples were reconstituted in 0.1% formic acid and centrifuged at 20,000× *g* for 10 min to remove insoluble debris. The resulting supernatants were subjected to quantitative proteomic analysis using a Vanquish Neo ultra-high-performance liquid chromatography (UHPLC) system coupled to an Orbitrap Astral mass spectrometer (Thermo Fisher Scientific, Waltham, MA, USA). Peptide separation was performed on an ES906 analytical column (150 mm) at a constant flow rate of 2.5 μL/min. Mobile phase A was 0.1% formic acid in ultrapure water, and mobile phase B consisted of 80% acetonitrile with 0.1% formic acid. The 8 min linear elution gradient was configured as follows: 4–25% mobile phase B from 0 to 4 min, 25–35% mobile phase B from 4 to 6.9 min, 35–99% mobile phase B from 6.9 to 7.3 min, and a final hold at 99% mobile phase B for 0.7 min (7.3–8.0 min) for column washing.

Mass spectral data were acquired in data-independent acquisition (DIA) mode with a precursor *m*/*z* range of 380–980. Full MS survey scans were collected at a resolution of 240,000, with an automatic gain control (AGC) target of 500% and a scan cycle time of 0.6 s. DIA fragmentation was implemented using 300 consecutive isolation windows with a width of 2 Th across the defined *m*/*z* range (380–980). The normalized collision energy (NCE) was set to 25%. The default precursor charge state was specified as +2, and the maximum injection time for fragment ion acquisition was limited to 3 ms.

All raw DIA mass spectrometry files were processed and analyzed using DIA-NN software (version 1.9.1) in library-free direct DIA analysis mode. Protein identification and quantification were performed against the *Mus musculus* reference protein sequence database downloaded from the UniProt database. Trypsin/P was designated as the digestion enzyme with a maximum of two missed cleavages allowed. Carbamidomethylation of cysteine was set as a fixed modification, while methionine oxidation and protein N-terminal acetylation were defined as variable modifications. The mass tolerance was set to 20 ppm for precursor ions and 0.05 Da for fragment ions. A target–decoy searching strategy was applied, and both peptide-spectrum match (PSM)-level and protein-level false discovery rate (FDR) were strictly controlled below 1%. Only protein groups with a protein-level FDR ≤ 1% were retained for subsequent quantitative and bioinformatic analysis.

### 2.8. Differential Protein Abundance Analysis

Protein abundance matrices generated by DIA-NN were exported as quantitative protein-intensity tables and further processed using R (version 4.4.1) for normalization, statistical analysis, and visualization. Protein intensity values were log_2_-transformed and median-normalized before statistical analysis. Proteins with missing values in more than 50% of the samples were excluded, and the remaining missing values were imputed using a minimum-value-based approach. Data quality and sample reproducibility were evaluated using protein abundance distributions, coefficients of variation, pairwise correlation analysis, and principal component analysis. All six proteomic samples were prepared and analyzed within the same analytical batch; therefore, no batch-correction procedure was applied.

Protein abundance differences between the melittin-treated and untreated control groups were evaluated using two-sided Student’s *t*-tests. Nominal *p* values were calculated for all quantified proteins. Proteins satisfying both a fold-change threshold of FC ≥ 1.20 or FC ≤ 0.83 and a nominal *p* < 0.05 were designated as candidate differentially abundant proteins (candidate DAPs) for exploratory downstream analysis.

Multiple-testing correction was not used to define the exploratory set of candidate differentially abundant proteins; therefore, no FDR-significant protein alterations were used to define the exploratory candidate set. Accordingly, these proteins are consistently referred to as candidate rather than FDR-significant differentially abundant proteins. Benjamini–Hochberg adjustment was applied to the subsequent enrichment analyses.

### 2.9. Functional Annotation of DAPs

Functional annotation was performed using the Gene Ontology (GO) (https://geneontology.org; accessed on 1 August 2026), Kyoto Encyclopedia of Genes and Genomes (KEGG), Reactome, and InterPro databases. Over-representation analysis was conducted using a hypergeometric test, with all reliably quantified proteins used as the background set. Enrichment *p* values were adjusted for multiple testing using the Benjamini–Hochberg method.

### 2.10. PPI Analysis of DAPs

Protein associations were retrieved from the STRING database (version 12.0) using *Mus musculus* as the reference organism. Associations with a combined confidence score ≥ 0.4 were retained and imported into Cytoscape (version 3.8.2) for network visualization and topological analysis. Network degree was calculated for each node, and proteins with relatively high degree values were prioritized as network-associated candidates. PPI analysis was performed to identify interaction networks among candidate proteins and prioritize highly connected network-associated candidates.

### 2.11. qPCR

RT-qPCR validation was performed to examine whether selected candidate protein-associated genes showed consistent transcriptional changes. The RT-qPCR validation targets were selected based on their biological relevance to oxidative stress and cellular stress adaptation, rather than solely according to network connectivity. Therefore, *Hspb1*, *Gch1*, and *Txnrd1* were selected as representative candidates associated with stress-response regulation.

Specific primers for *Hspb1*, *Gch1*, and *Txnrd1* were designed using Primer-BLAST (NCBI, National Center for Biotechnology Information, Bethesda, MD, USA; https://www.ncbi.nlm.nih.gov/tools/primer-blast/; accessed on 23 September 2026) and synthesized by Sangon Biotech (Shanghai, China). Primer sequences are listed in Table 1. Total RNA was extracted from E0771 cells using TRIzol reagent (Yeasen Biotechnology, Shanghai, China) and reverse-transcribed into cDNA using the Hifair^®^ III 1st Strand cDNA Synthesis SuperMix kit (Yeasen Biotechnology, Shanghai, China). Quantitative real-time PCR was performed using Hieff^®^ qPCR SYBR Green Master Mix (Yeasen Biotechnology, Shanghai, China) on a real-time fluorescence quantitative PCR system (TIANLONG, Xi’an, China). The amplification conditions were as follows: initial denaturation at 95 °C for 30 s, followed by 40 cycles of 95 °C for 10 s and 60 °C for 30 s. The relative expression levels of target genes were normalized to *actin* and calculated using the 2^−ΔΔCt^ method. Each reaction was performed with three technical replicates, and experiments were independently repeated three times. Statistical analysis was conducted using one-way analysis of variance (ANOVA) followed by Tukey’s multiple comparison test. Statistical significance was defined as ns, *p* > 0.05; *, *p* < 0.05; **, *p* < 0.01; ***, *p* < 0.001; and ****, *p* < 0.0001. Data visualization was performed using GraphPad Prism 10.0.

## 3. Results

### 3.1. Overall Experimental Workflow

The overall experimental workflow is shown in Figure 1 E0771 cells were treated with melittin and evaluated using colony formation and CCK-8 assays. Proteins were then extracted and analyzed by DIA-based proteomics, followed by quality control, differential protein analysis, functional enrichment, protein–protein interaction analysis, and RT–qPCR validation.

### 3.2. Effects of Melittin on the Colony-Forming Capacity and Viability of E0771 Cells

To evaluate the effects of melittin on the long-term proliferative capacity and viability of E0771 cells, colony formation and CCK-8 assays were performed following treatment with increasing concentrations of melittin. As shown in Figure 2A, numerous crystal violet-stained colonies were observed in the untreated control group. With increasing melittin concentrations, the number and overall size of the colonies progressively decreased. E0771 cells treated with 1 μg/mL melittin retained a relatively large number of colonies, whereas colony formation was visibly reduced following treatment with 2 and 4 μg/mL melittin. At 8 μg/mL, almost no colonies were detected.

Quantitative analysis showed that the colony number was lower in the 1 μg/mL melittin-treated group than in the untreated control group, although the difference was not statistically significant. Treatment with 2 and 4 μg/mL melittin significantly reduced the number of colonies, while 8 μg/mL melittin almost completely abolished colony formation (Figure 2B).

The CCK-8 assay further showed a progressive reduction in E0771 cell viability with increasing melittin concentrations. Compared with the untreated control group, cell viability was significantly reduced following treatment with 1 μg/mL melittin and decreased further at 2, 4, and 8 μg/mL (Figure 2C). Collectively, these results showed that melittin reduced both the colony-forming capacity and viability of E0771 cells in a concentration-dependent manner. To further characterize the early molecular responses associated with these phenotypic effects, we next performed DIA-based quantitative proteomic profiling using E0771 cells treated with 4 μg/mL melittin for 15 min and matched untreated controls, with three biological replicates per group.

### 3.3. Quality Control of Proteomic Experimental Samples

To characterize the overall data distribution and sample-to-sample consistency of the proteomic dataset, protein abundance distributions and pairwise correlation analyses were performed across all experimental samples. As shown in Figure 3A, the log_2_-transformed protein expression values of the six samples, including Ctr-1, Ctr-2, Ctr-3, Mel-1, Mel-2, and Mel-3, displayed comparable distribution patterns. The violin plots showed similar overall shapes among samples, and the median values in the embedded boxplots were closely aligned, with the majority of quantified proteins distributed within a comparable abundance range.

Pearson correlation coefficients were further calculated based on the protein quantification matrix. As shown in Figure 3B, the pairwise correlation coefficients among the six samples ranged from 0.96 to 0.99 (Figure 3B). Within the Ctr group, the correlation coefficients ranged from 0.98 to 0.99, whereas those within the Mel group were 0.99. The correlation coefficients between Ctr and Mel samples ranged from 0.96 to 0.99. In the correlation plot, the upper triangle displays the numerical correlation coefficients, while the lower triangle represents the correlation strength using circle size and color intensity, together with significance annotations. The source data supporting the quality control assessment, including the protein identification summary, sample information, peptide sequence length range, and candidate differential protein statistics, are provided in Table 2.

### 3.4. Analysis of DEPs in E0771 Cells Following Melittin Treatment

Comparative analysis identified a total of 9748 quantifiable proteins. Among these, there were 339 DEPs, including 114 up-regulated and 225 down-regulated proteins (Figure 4). Further investigation indicated that the number of down-regulated candidates was greater than that of up-regulated candidates. The top 20 most significantly DEPs (ranked by *p* value) were labeled in Figure 4.

KEGG pathway enrichment analysis demonstrated that the top 20 enriched KEGG pathways by DEPs mainly covered three core categories, namely cell junction and cytoskeleton regulation, key tumor signaling pathways, and immune inflammation and metabolism (Figure 5A). Specifically, the cell junction and cytoskeleton regulation category included gap junction, focal adhesion, tight junction, and regulation of actin cytoskeleton. The key tumor signaling pathways comprised Hippo, VEGF, ErbB, and apoptosis. The immune inflammation and metabolism category contained T cell receptor signaling pathway, Fc epsilon RI signaling pathway, and Mucin type O-glycan biosynthesis, among others. Detailed information regarding all KEGG pathways is shown in Table 3.

GO enrichment analysis classified the terms displayed in Figure 5B into the three major ontological categories of Biological Process, Cellular Component, and Molecular Function. Within the Biological Process category, the candidate differentially abundant proteins were enriched in phagocytosis, energy homeostasis, regulation of cell shape, insulin receptor signaling pathway, anterior commissure morphogenesis, response to calcium ion, positive regulation of apoptotic process, regulation of cell population proliferation, positive regulation of TORC1 signaling, and cytoskeleton organization.

Within the Cellular Component category, the enriched terms included cytosol, cytoplasm, nucleoplasm, mitochondrion, lamellipodium, focal adhesion, nucleus, Z disk, endosome, and Golgi membrane. Within the Molecular Function category, the enriched terms comprised identical protein binding, protein serine kinase activity, structural constituent of cytoskeleton, enzyme binding, protein kinase binding, protein kinase C binding, GTPase activator activity, calcium-dependent phospholipid binding, calcium-dependent protein binding, and beta-D-galactosyl-(1→3)-N-acetyl-beta-D-galactosaminide alpha-2,3-sialyltransferase activity (Figure 5B). The representative GO terms displayed in Figure 5B are listed in Table 4.

### 3.5. PPI Network of DEPs

To summarize the STRING-derived protein associations among candidate differentially abundant proteins, a protein association network was constructed and visualized using Cytoscape (version 3.8.2), which included 132 nodes and 216 association edges (Figure 6). Proteins with relatively high degree values included CASP8, RPS27A, ANXA5, PIK3CA, MSN, MAP2K7, PXN, JAK1, TSG101, and YWHAG. CASP8 had the highest degree value of 17, followed by RPS27A with 14, whereas ANXA5 and PIK3CA each had a degree value of 13. The STRING combined scores of the retained associations ranged from 0.400 to 0.999. According to STRING interaction scores, the 216 edges had scores ranging from 400 to 999. Among them, 157 interactions were classified as medium confidence, 33 as high confidence, and 26 as highest confidence.

### 3.6. RT-qPCR Validation of DEP-Encoding Genes

To verify the reliability of the quantitative proteomic results, the genes encoding three differentially expressed proteins—*Hspb1*, *Gch1*, and *Txnrd1*—were selected for RT–qPCR analysis in murine E0771 breast cancer cells. Compared with the control group, melittin treatment markedly reduced the relative mRNA expression of *Hspb1* (Figure 7A, *p* < 0.01). The expression levels of *Gch1* and *Txnrd1* were also significantly decreased following melittin treatment (Figure 7B, *p* < 0.01; Figure 7C, *p* < 0.05). These transcriptional changes were consistent with the expression trends of the corresponding proteins identified by quantitative proteomic analysis, supporting the reliability of the proteomic dataset.

## 4. Discussion

Breast cancer comprises molecularly heterogeneous diseases with distinct biological characteristics and therapeutic responses. Although multiple treatment modalities have improved clinical outcomes, therapeutic resistance and disease recurrence remain major challenges. Although chemotherapy, immunotherapy, PARP inhibitors, and antibody–drug conjugates have expanded the therapeutic landscape, treatment resistance and recurrence remain major clinical problems [19,20]. Melittin, the principal amphipathic peptide of honeybee venom, has been reported to exert antitumor activity in breast cancer models, including aggressive breast cancer subtypes, partly through disruption of membrane integrity and modulation of receptor tyrosine kinase-associated signaling [4,21]. Recent studies have emphasized that melittin-induced anticancer effects may involve multiple interconnected cellular processes rather than a single signaling pathway, including membrane perturbation, stress responses, metabolic alterations, and cell-death-associated signaling [22]. In the present study, melittin treatment reduced both the colony-forming capacity and CCK-8-determined viability of E0771 cells in a concentration-dependent manner and was followed by DIA-based quantitative proteomic profiling. These results provide a proteomic view of early cellular responses to melittin in murine breast cancer cells.

The quality-control analyses showed comparable log_2_-transformed protein abundance distributions and high pairwise correlations among biological replicates, supporting the technical consistency of the proteomic dataset (Figure 3). On basis of predefined fold-change and nominal *p* value thresholds, 339 DEPs were identified after melittin treatment. Since the current screening strategy is based on nominal statistical significance, these proteins should be interpreted as candidate abundance changes rather than definitive FDR-significant protein alterations. This distinction is important because proteomic discovery studies are powerful for generating mechanistic hypotheses, but candidate proteins generally require orthogonal validation by targeted proteomics, immunoblotting, or functional assays before being assigned causal roles [23].

Functional enrichment analysis indicated that melittin-responsive candidate proteins were associated with cell junctions, focal adhesion, tight junctions, and regulation of the actin cytoskeleton. These categories are biologically relevant because cell adhesion and cytoskeletal organization are closely linked to cell shape, mechanical stability, migration, and invasive behavior in cancer cells [24]. These enrichment results are best interpreted as evidence that melittin treatment is accompanied by proteomic alterations in structural and adhesion-related protein networks, rather than as direct proof that melittin inhibits metastatic behavior in this model. Taken together, these findings suggest that short-term melittin exposure may induce coordinated early changes involving membrane-associated structures, cytoskeletal organization, signaling, and mitochondrial responses [22]. Notably, 31 candidate proteins were annotated to the mitochondrial compartment. However, since ROS production and mitochondrial function were not directly assessed, mitochondrial involvement should be regarded as a hypothesis requiring further validation.

Several nominally enriched pathways were relative to growth-factor or survival signaling, including ErbB, VEGF, FoxO, and PI3K-associated signaling contexts. This observation is consistent with previous evidence that honeybee venom and melittin can suppress EGFR and HER2 phosphorylation and dynamically alter downstream signaling in HER2-enriched and triple-negative breast cancer cells [21]. Among the high-connectivity nodes in the PPI network, PIK3CA is particularly relevant because the PI3K/AKT pathway regulates multiple cancer-associated phenotypes, including cell survival, proliferation, metabolism, angiogenesis, and therapy resistance [25]. Nevertheless, protein abundance changes in PIK3CA or enrichment of related pathways do not necessarily demonstrate altered pathway activity. Future experiments assessing phosphorylated AKT, mTOR, FOXO, or upstream receptor activation would be required to determine whether PI3K/AKT signaling is functionally inhibited under the present treatment conditions.

The enrichment of apoptosis-related pathways and the high network connectivity of CASP8 are compatible with the observed reduction in clonogenic survival after melittin exposure. CASP8 is classically involved in death receptor-mediated apoptosis, but it also has context-dependent non-apoptotic roles in inflammation, autophagy, migration, and tumor biology [26]. Hence, the identification of CASP8 as a high-degree node supports its prioritization as a candidate for validation, but does not by itself establish caspase-8-dependent apoptosis as the dominant mechanism. In parallel, ANXA5 was also identified as a high-connectivity candidate. Annexin A5 binds to phosphatidylserine-exposing membranes and has been widely used or investigated in relation to apoptotic membrane remodeling and tumor-targeting strategies [27,28]. Together, CASP8 and ANXA5 provide a biologically plausible link between melittin-induced membrane perturbation and cell-death-associated responses. However, this relationship remains to be experimentally tested.

The PPI network further prioritized CASP8, RPS27A, ANXA5, PIK3CA, MSN, MAP2K7, PXN, JAK1, TSG101, and YWHAG as relatively high-connectivity proteins. In interpreting these results, it is important to note that STRING integrates experimentally supported interactions, computational predictions, curated knowledge, and functional associations. Therefore, STRING-derived edges should not be interpreted as evidence of direct physical binding for every protein pair [29]. Cytoscape-based network visualization is useful for organizing candidate proteins and identifying highly connected nodes [30], but network degree alone does not establish regulatory hierarchy. Accordingly, CASP8, PIK3CA, and ANXA5 are better described as network-prioritized candidates for validation rather than as a validated CASP8-PIK3CA-ANXA5 regulatory axis. Beyond CASP8, RPS27A, ANXA5, and PIK3CA, several additional high-connectivity proteins identified from the PPI network may also contribute to the cellular responses induced by melittin. MSN and PXN are associated with cytoskeletal organization and cell adhesion processes, which is consistent with the enrichment of actin cytoskeleton and focal adhesion-related pathways observed in this study [31]. MAP2K7 and JAK1 are involved in stress-responsive and cytokine-associated signaling regulation, suggesting potential roles in adaptive cellular responses following melittin exposure [32,33]. TSG101 and YWHAG have been implicated in intracellular trafficking, signal modulation, and regulation of cellular homeostasis [34]. RPS27A, a ribosomal ubiquitin fusion protein, may reflect alterations in protein synthesis and stress adaptation programs [35]. However, because these candidates were prioritized based on network connectivity rather than direct functional validation, their specific contributions to melittin-induced anticancer responses remain to be experimentally determined.

In addition to the up-regulated candidate proteins, the larger proportion of down-regulated proteins identified following melittin exposure might also contribute to the observed anticancer phenotype. Reduced abundance of these proteins may reflect suppression of cellular programs engaged in proliferation, survival, cytoskeletal maintenance, and stress adaptation. Consistent with this possibility, enrichment analysis indicated that candidate proteins were associated with regulation of cell population proliferation, cytoskeleton organization, and apoptosis-related processes. However, whether individual down-regulated protein directly mediate melittin-induced inhibition of breast cancer cell growth or influence tumor-associated immune interactions requires further functional investigation.

Several limitations should be taken into consideration. First, the study was performed using a single murine mammary carcinoma cell line, which limited the generalizability of the findings to other breast cancer models. Second, the proteomic analysis included three biological replicates per group, limiting statistical power for high-dimensional differential-abundance analysis. Third, the 15 min melittin exposure was designed to characterize early cellular responses and may not capture later molecular events related to sustained cytotoxicity. In addition, pathway enrichment and PPI analyses are exploratory and do not establish causal relationships. RT-qPCR validation was limited to *Hspb1*, *Gch1*, and *Txnrd1* and provides transcript-level support rather than direct protein-level validation. The functional roles of CASP8, PIK3CA, ANXA5, and RPS27A also remain to be experimentally confirmed.

Future studies should determine whether these early proteomic responses are reproducible in human breast cancer models with different molecular backgrounds. Protein-level and functional validation will also be required to clarify whether network-prioritized candidates such as CASP8, PIK3CA, ANXA5, and RPS27A contribute causally to melittin-induced cellular responses. Time-course and dose–response studies, together with three-dimensional models, breast cancer organoids, and in vivo systems, may further help define the temporal sequence of signaling, mitochondrial, and cell-death-associated events and evaluate the antitumor efficacy and safety of melittin-based strategies.

## 5. Conclusions

In conclusion, short-term melittin exposure reduced the clonogenic capacity and viability of E0771 murine mammary carcinoma cells and was associated with broad candidate protein-abundance changes involving cellular structure, signaling, metabolism, and cell-death-associated processes. DIA-based proteomic analysis further prioritized CASP8, RPS27A, ANXA5, and PIK3CA as candidate proteins for follow-up investigation. These findings provide a proteomic basis for understanding early cellular responses to melittin but should be interpreted as hypothesis-generating rather than causal mechanistic evidence.

## Figures and Tables

**Figure 1:**
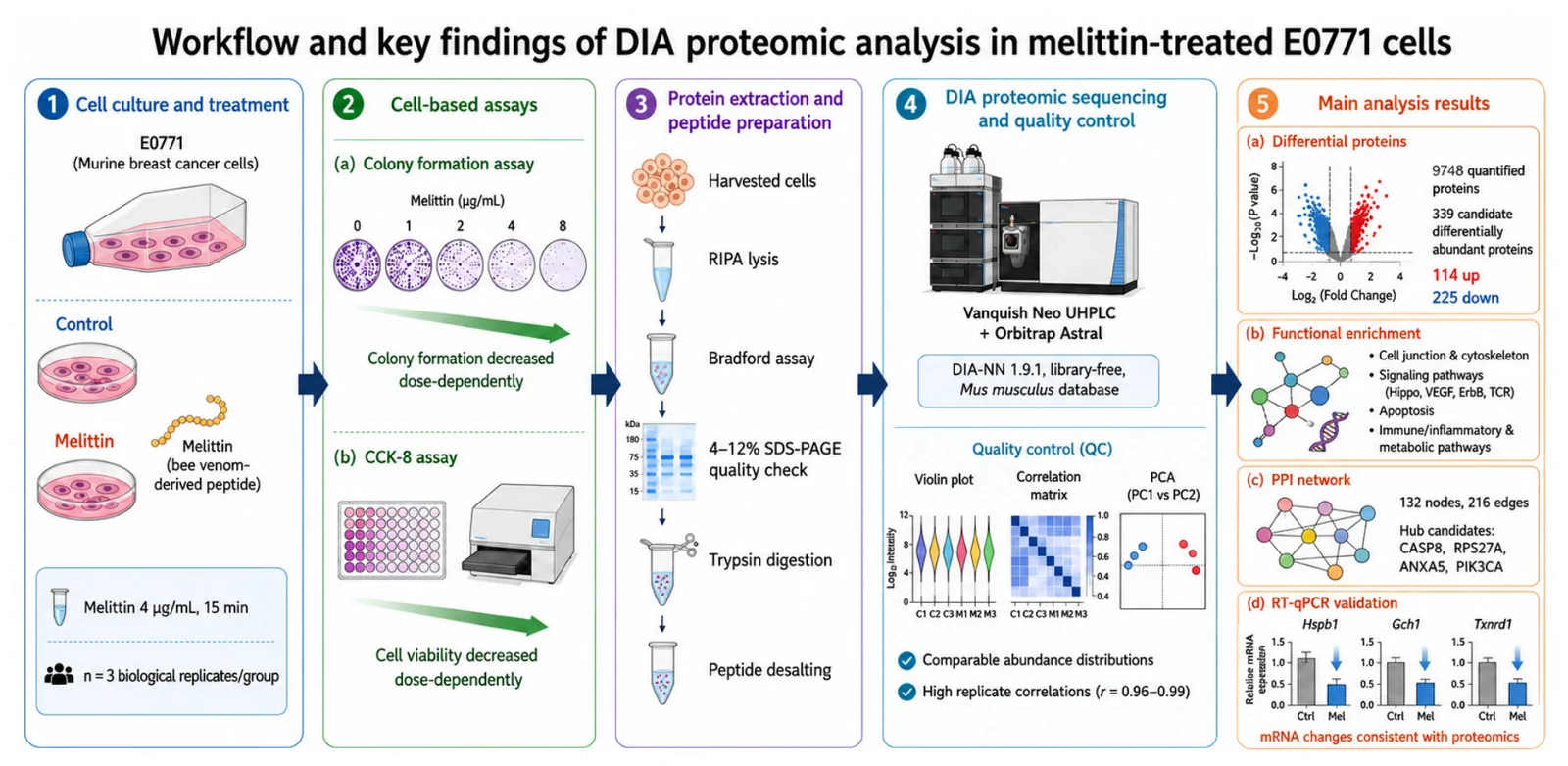
Overall experimental workflow of this study.

**Figure 2:**
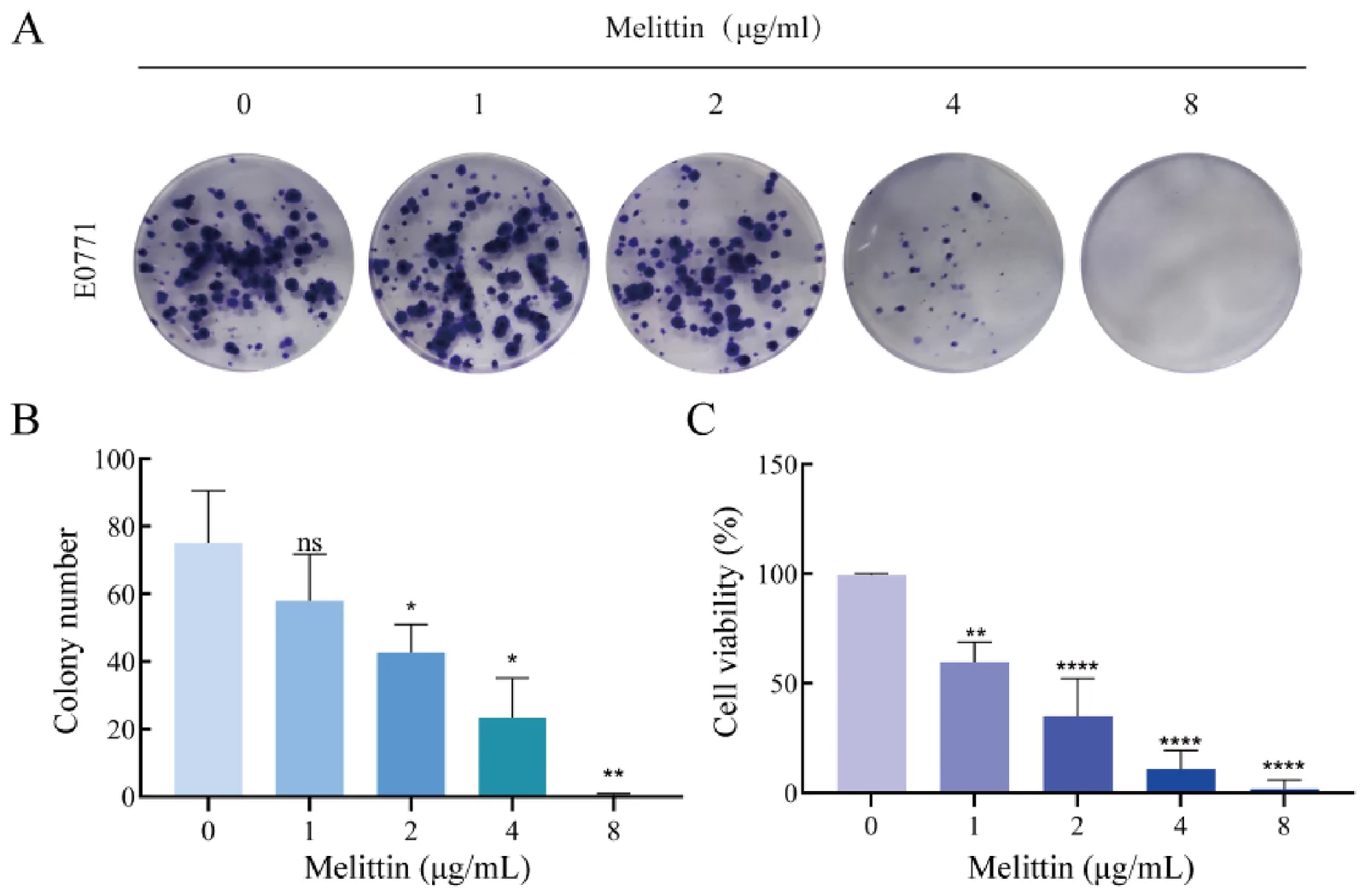
Effects of melittin on the colony-forming capacity and viability of E0771 cells. (**A**) Representative images of colonies formed by E0771 cells following treatment with 0, 1, 2, 4, or 8 μg/mL melittin. (**B**) Quantification of colony numbers in E0771 cells treated with the indicated concentrations of melittin. (**C**) Cell viability of E0771 cells following melittin treatment, as determined using the CCK-8 assay. Data are presented as the mean ± SD. Statistical significance was assessed relative to the untreated control group. ns, not significant; * *p* < 0.05; ** *p* < 0.01; **** *p* < 0.0001. Statistical significance was evaluated using one-way ANOVA. *n* = 3 independent experiments.

**Figure 3:**
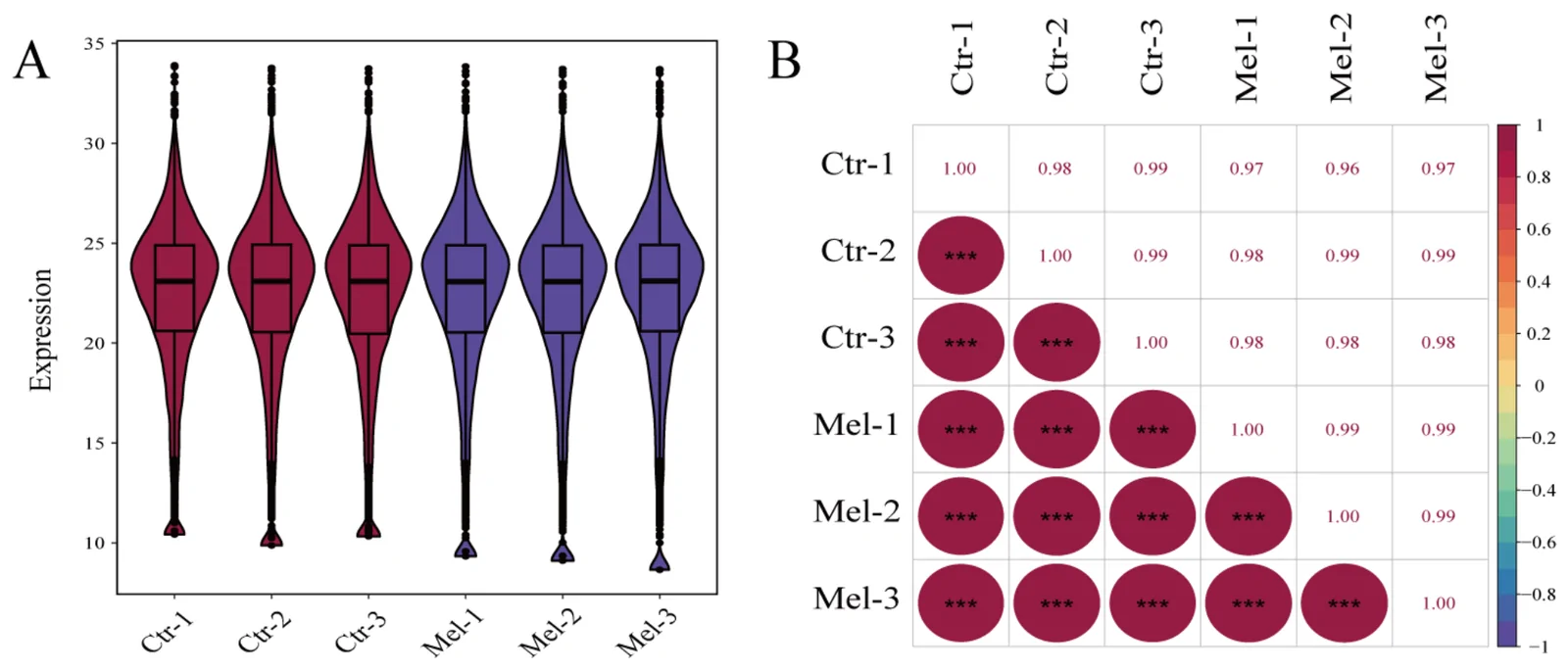
Distribution and correlation analysis of proteomic experimental samples. (**A**) Violin plot showing the distribution of log_2_-transformed protein expression values across Ctr and Mel samples. Boxplots are embedded within the violin plots to indicate the median and interquartile range. (**B**) Pearson correlation analysis among proteomic samples based on the protein quantification matrix. The upper triangle shows the correlation coefficients, whereas the lower triangle represents the correlation strength using circle size and color intensity. Significance levels are indicated by asterisks. Ctr-1, Ctr-2, and Ctr-3 represent three independent biological replicates of the untreated control group; Mel-1, Mel-2, and Mel-3 represent three independent biological replicates of E0771 cells treated with 4 μg/mL melittin for 15 min. *** *p* < 0.001.

**Figure 4:**
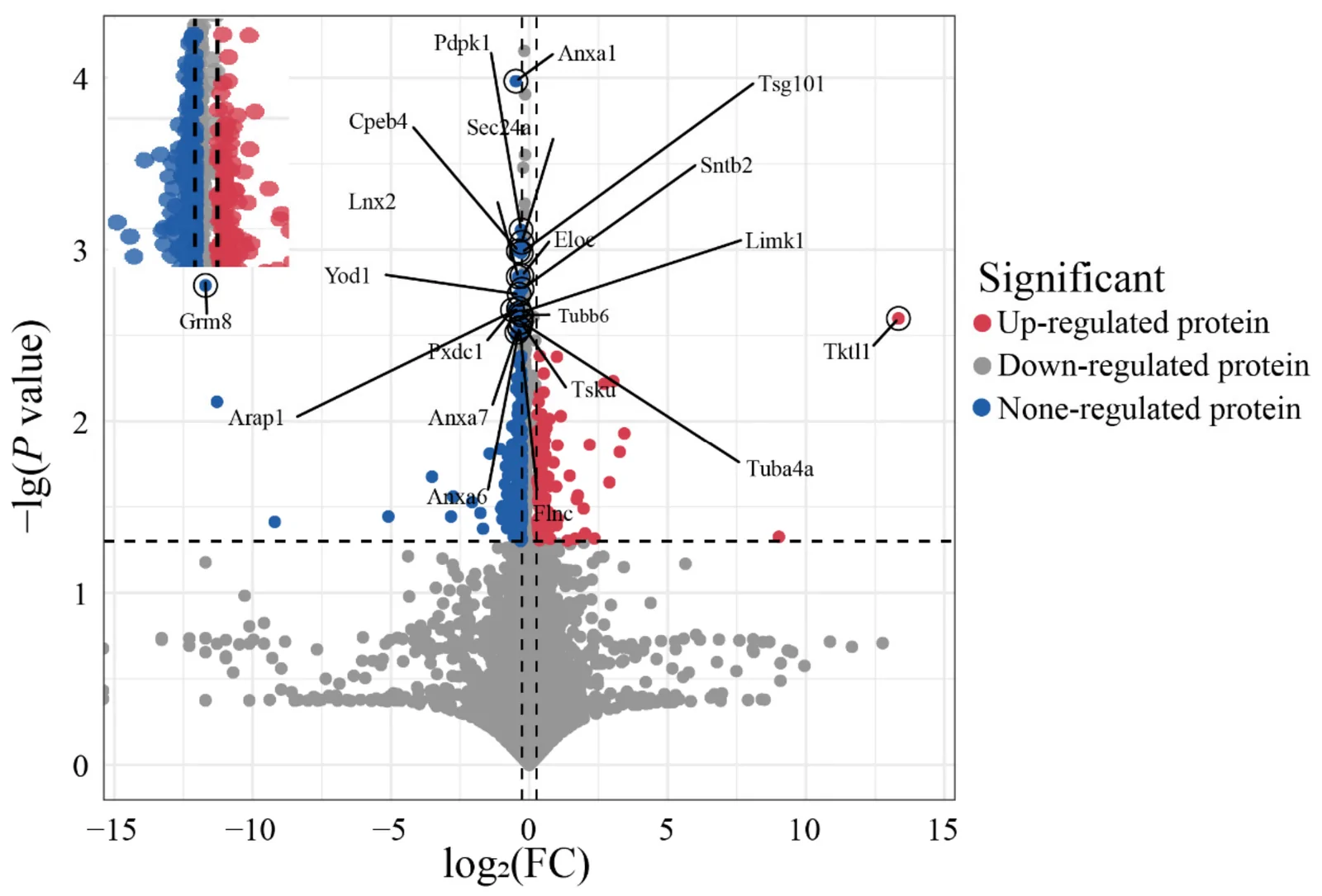
Volcano plot of differentially expressed proteins in E0771 murine breast cancer cells. following melittin treatment. Down-regulated proteins: P47743 (Grm8), P14824 (Anxa6), Q8CB27 (Yod1), Q7TN98 (Cpeb4), Q9Z2A0 (Pdpk1), Q91XL2 (Lnx2), Q4LDD4 (Arap1), Q8JZU6 (Pxdc1), Q07076 (Anxa7). Up-regulated proteins: P10107 (Anxa1), Q3U2P1 (Sec24a), P83940 (Eloc), G922E4 (Tubb6), Q8CBR6 (Tsku), Q8VHX6 (Flnc), Q61187 (Tsg101), Q61235 (Sntb2), P53668 (Limk1), P68368 (Tuba4a), Q99MX0 (Tktl1).

**Figure 5:**
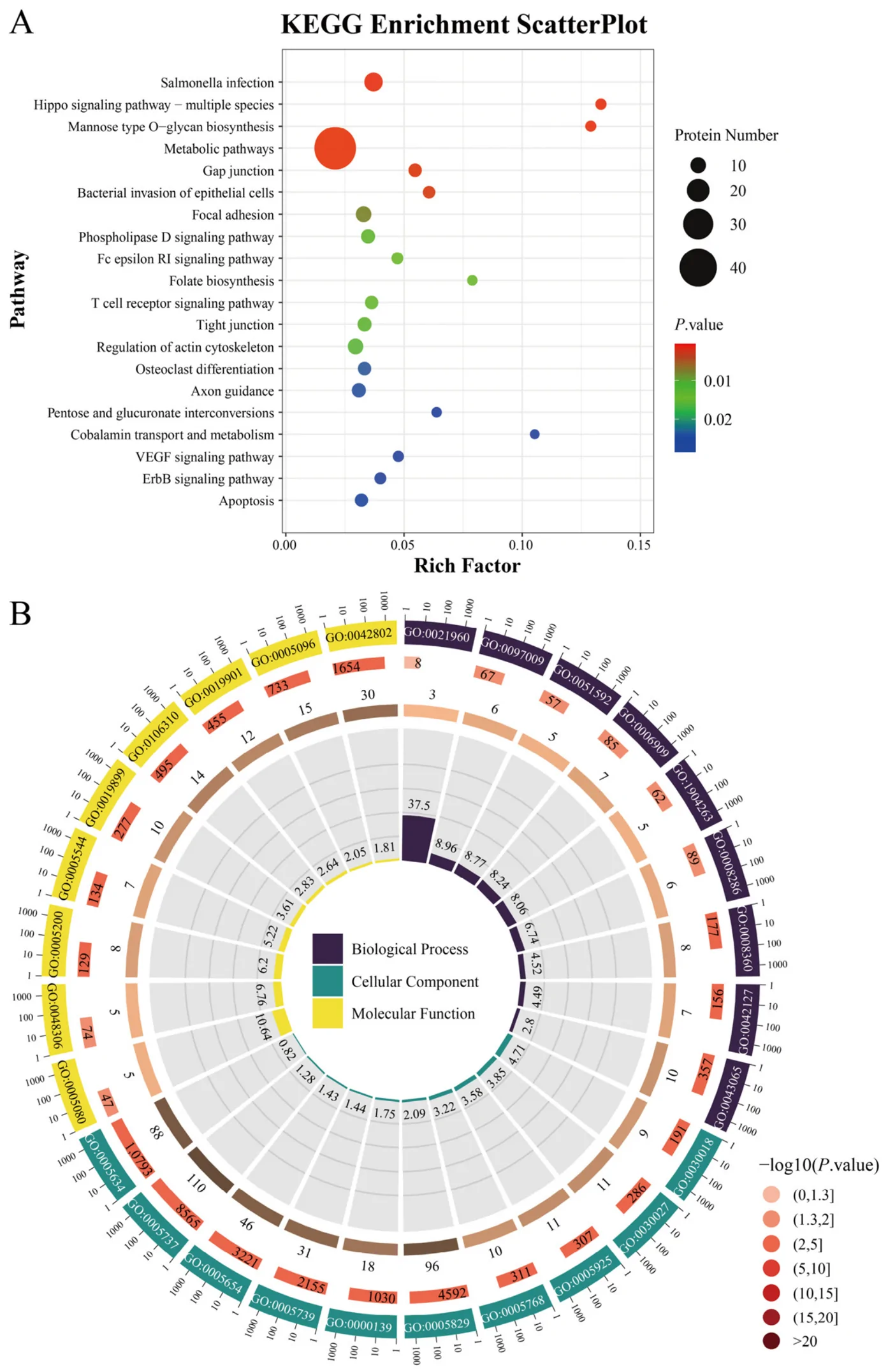
KEGG pathways and GO terms significantly enriched by differentially expressed proteins in melittin-treated E0771 murine breast cancer cells. (**A**) KEGG pathway enrichment scatter plot. The x-axis represents the Rich factor, bubble size indicates the number of candidate proteins assigned to each pathway, and bubble color represents the nominal *p* value. (**B**) Circular representation of representative GO enrichment terms classified into Biological Process, Cellular Component, and Molecular Function categories. The concentric tracks display GO identifiers, candidate protein numbers, enrichment ratios, and nominal enrichment significance.

**Figure 6:**
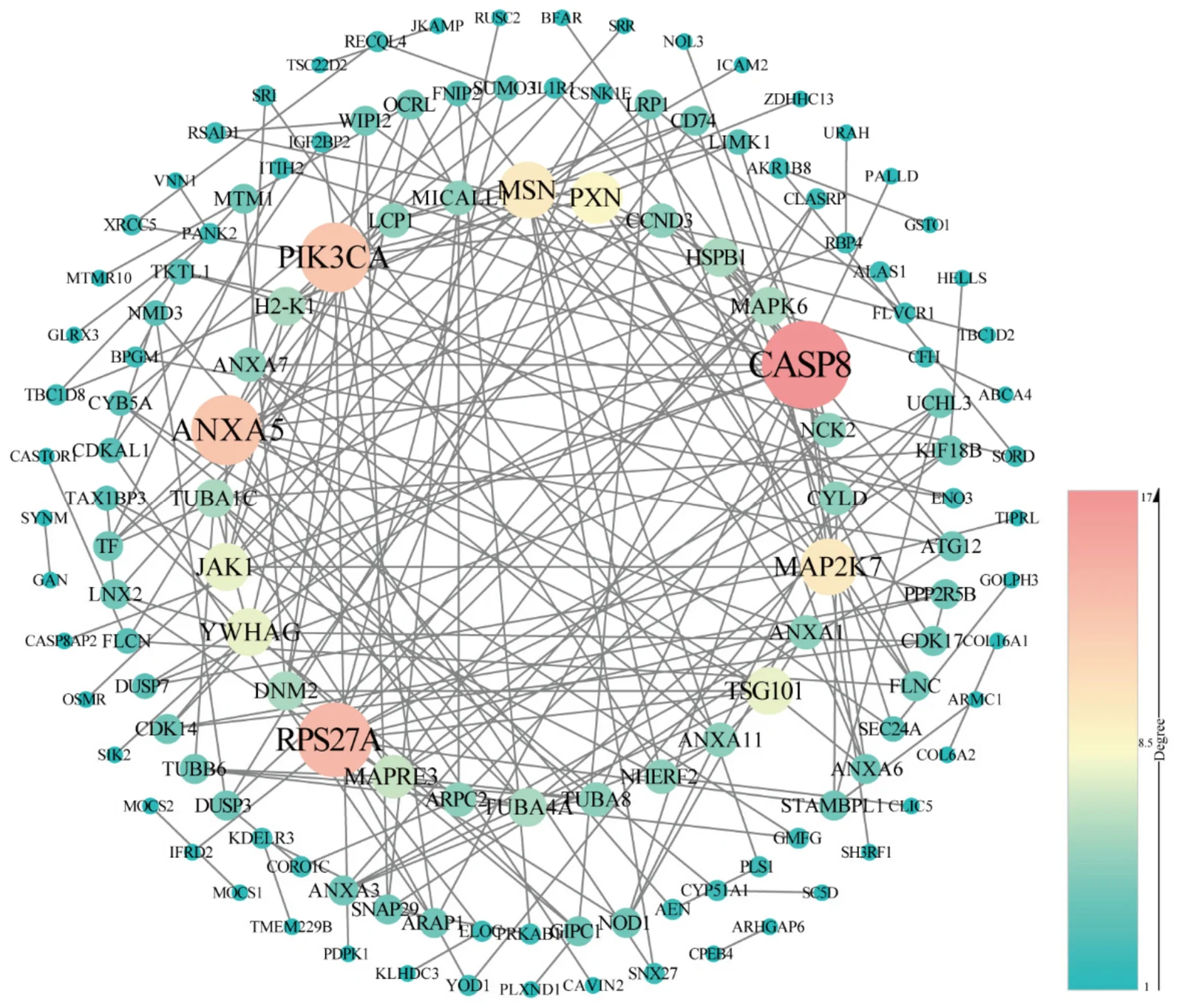
Protein–protein interaction network analysis of differentially expressed proteins. Each node represents a candidate differential protein, and each edge represents a known or predicted protein association recorded in the STRING database. Node size and color were mapped according to network degree, with proteins showing higher connectivity displayed as larger nodes.

**Figure 7:**
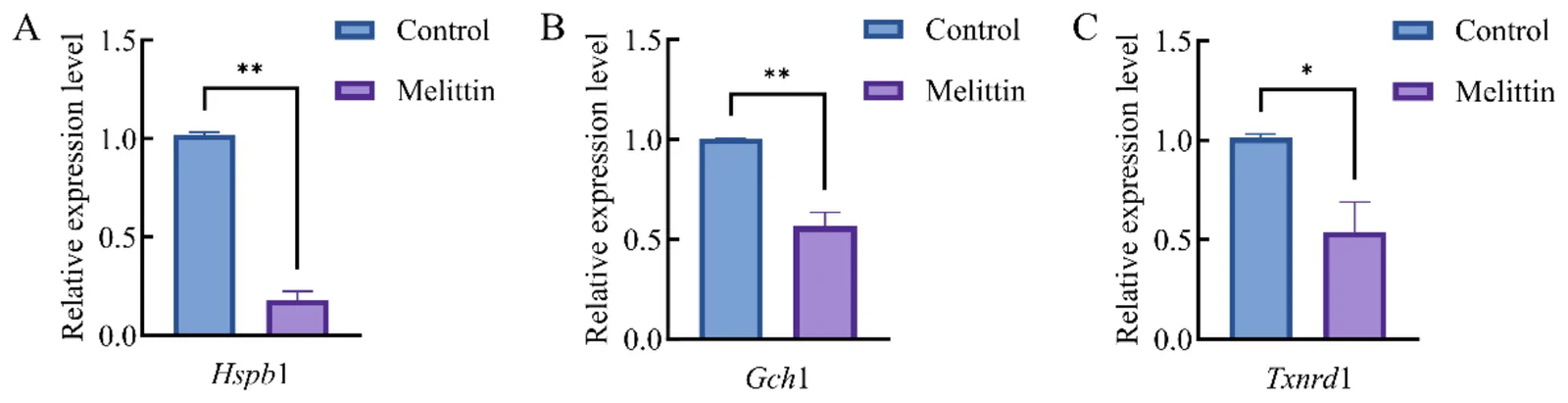
RT–qPCR validation of selected differentially expressed protein-encoding genes in E0771 cells. Relative mRNA expression levels of (**A**) *Hspb1*, (**B**) *Gch1*, and (**C**) *Txnrd1* in control and melittin-treated E0771 murine breast cancer cells. Gene expression in the control group was normalized to 1. Data are presented as the mean ± SD. * *p* < 0.05 and ** *p* < 0.01 versus the control group.

**Table 1:** Primer sequences used for RT–qPCR analysis.

GenBank Accession	Gene	Forward Primer (5′–3′)	Reverse Primer (5′–3′)
NM_013560.2	*Hspb1*	GAGATCACCGGCATCGTCT	TCTCCAGTCTTCGTTGGCTC
NM_008102.4	*Gch1*	GCTGCTGCTGTTCTACCTGA	TGGTCCAGGTAGATGGTGGT
NM_001042523.1	*Txnrd1*	AGGACCTGCCTTCTGTTGGT	TCTGGGTTCTCCAGCTTCTG
NM_007393.5	*actin*	GGCTGTATTCCCCTCCATCG	CCAGTTGGTAACAATGCCATGT

**Table 2:** Summary of protein identification and quality control metrics for DIA-based proteomics.

Metric	Value
Quantified protein entries retained for analysis	9748
Samples included in the analysis	6
Control samples	3
Melittin-treated samples	3
Peptide sequence length range	7–37 aa
Proteins with nominal *p* value < 0.05	728
Candidate differentially abundant proteins	339
Up-regulated candidate differentially abundant proteins	114
Down-regulated candidate differentially abundant proteins	225

aa = amino acid residues.

**Table 3:** Enriched KEGG pathways displayed for candidate differentially expressed proteins in melittin-treated E0771 cells.

Pathway ID	Pathway Name	Protein Number	Rich Factor	*p* Value
mmu05132	Salmonella infection	14	0.0371	0.000559012
mmu04392	Hippo signaling pathway-multiple species	4	0.1333	0.000655961
mmu00515	Mannose type O-glycan biosynthesis	4	0.129	0.00074532
mmu01100	Metabolic pathways	46	0.0209	0.00100546
mmu04540	Gap junction	7	0.0547	0.001687947
mmu05100	Bacterial invasion of epithelial cells	6	0.0606	0.002150383
mmu04510	Focal adhesion	10	0.0329	0.00798051
mmu04072	Phospholipase D signaling pathway	8	0.0348	0.012484007
mmu04664	Fc epsilon RI signaling pathway	5	0.0472	0.014080296
mmu00790	Folate biosynthesis	3	0.0789	0.014248538
mmu04660	T cell receptor signaling pathway	7	0.0363	0.015415957
mmu04530	Tight junction	8	0.0333	0.015779772
mmu04810	Regulation of actin cytoskeleton	10	0.0295	0.016212741
mmu04380	Osteoclast differentiation	7	0.0333	0.023304611
mmu04360	Axon guidance	8	0.0309	0.023709525
mmu00040	Pentose and glucuronate interconversions	3	0.0638	0.02509837
mmu04980	Cobalamin transport and metabolism	2	0.1053	0.026428116
mmu04370	VEGF signaling pathway	4	0.0476	0.026455128
mmu04012	ErbB signaling pathway	5	0.04	0.026658172
mmu04210	Apoptosis	7	0.032	0.028447137
mmu04068	FoxO signaling pathway	7	0.0317	0.029686644
mmu04670	Leukocyte transendothelial migration	6	0.0345	0.030017923
mmu05418	Fluid shear stress and atherosclerosis	7	0.0311	0.03227424
mmu05020	Prion disease	10	0.0263	0.032446887
mmu00533	Glycosaminoglycan biosynthesis-keratan sulfate	2	0.0909	0.034779596
mmu00051	Fructose and mannose metabolism	3	0.0556	0.035886548
mmu00260	Glycine, serine and threonine metabolism	3	0.0545	0.03759365
mmu05231	Choline metabolism in cancer	5	0.0345	0.04590755
mmu00514	Other types of O-glycan biosynthesis	3	0.05	0.04673858
mmu04960	Aldosterone-regulated sodium reabsorption	3	0.0492	0.048687647

**Table 4:** Representative GO terms identified by enrichment analysis of candidate differentially expressed proteins in melittin-treated E0771 cells.

GO Category	GO ID	GO Term	Protein Number
Biological Process	GO:0006909	Phagocytosis	7
Biological Process	GO:0097009	Energy homeostasis	6
Biological Process	GO:0008360	Regulation of cell shape	8
Biological Process	GO:0008286	Insulin receptor signaling pathway	6
Biological Process	GO:0021960	Anterior commissure morphogenesis	3
Biological Process	GO:0051592	Response to calcium ion	5
Biological Process	GO:0043065	Positive regulation of apoptotic process	10
Biological Process	GO:0042127	Regulation of cell population proliferation	7
Biological Process	GO:1904263	Positive regulation of TORC1 signaling	5
Biological Process	GO:0007010	Cytoskeleton organization	7
Cellular Component	GO:0005829	Cytosol	96
Cellular Component	GO:0005737	Cytoplasm	110
Cellular Component	GO:0005654	Nucleoplasm	46
Cellular Component	GO:0005739	Mitochondrion	31
Cellular Component	GO:0030027	Lamellipodium	11
Cellular Component	GO:0005925	Focal adhesion	11
Cellular Component	GO:0005634	Nucleus	88
Cellular Component	GO:0030018	Z disk	9
Cellular Component	GO:0005768	Endosome	10
Cellular Component	GO:0000139	Golgi membrane	18
Molecular Function	GO:0042802	Identical protein binding	30
Molecular Function	GO:0106310	Protein serine kinase activity	14
Molecular Function	GO:0005200	Structural constituent of cytoskeleton	8
Molecular Function	GO:0019899	Enzyme binding	10
Molecular Function	GO:0019901	Protein kinase binding	12
Molecular Function	GO:0005080	Protein kinase C binding	5
Molecular Function	GO:0005096	GTPase activator activity	15
Molecular Function	GO:0005544	Calcium-dependent phospholipid binding	7
Molecular Function	GO:0048306	Calcium-dependent protein binding	5
Molecular Function	GO:0047288	Beta-D-galactosyl-(1→3)-N-acetyl-beta-D-galactosaminide alpha-2,3-sialyltransferase activity	2

## Data Availability

The raw mass spectrometry data and processed proteomic datasets generated and analyzed during the current study have not been deposited in a public repository. These data are available from the corresponding authors upon reasonable request.

## Abbreviations

The following abbreviations are used in this manuscript:

ANOVA	Analysis of variance
BH	Benjamini–Hochberg
BH4	Tetrahydrobiopterin
CCK-8	Cell Counting Kit-8
DAP	Differentially abundant protein
DIA	Data-independent acquisition
DIA-NN	Data-independent acquisition by neural networks
DMEM	Dulbecco’s modified Eagle medium
EGFR	Epidermal growth factor receptor
FBS	Fetal bovine serum
FC	Fold change
FDR	False discovery rate
GO	Gene Ontology
HER2	Human epidermal growth factor receptor 2
KEGG	Kyoto Encyclopedia of Genes and Genomes
LC–MS/MS	Liquid chromatography–tandem mass spectrometry
MRM	Multiple reaction monitoring
mTOR	Mechanistic target of rapamycin
PBS	Phosphate-buffered saline
PCA	Principal component analysis
PI3K/AKT	Phosphoinositide 3-kinase/protein kinase B
PPI	Protein–protein interaction
PRM	Parallel reaction monitoring
qPCR	Quantitative polymerase chain reaction
RIPA	Radioimmunoprecipitation assay
ROS	Reactive oxygen species
RT-qPCR	Reverse-transcription quantitative polymerase chain reaction
SD	Standard deviation
SDS–PAGE	Sodium dodecyl sulfate–polyacrylamide gel electrophoresis
STRING	Search Tool for the Retrieval of Interacting Genes/Proteins
STR	Short tandem repeat
TNBC	Triple-negative breast cancer
TORC1	Target of rapamycin complex 1
UHPLC	Ultra-high-performance liquid chromatography
VEGF	Vascular endothelial growth factor

## References

[1] Ge L.P., Jin X., Ma D., Wang Z.Y., Liu C.L., Zhou C.Z., Zhao S., Yu T.J., Liu X.Y., Di G.H. (2024). ZNF689 deficiency promotes intratumor heterogeneity and immunotherapy resistance in triple-negative breast cancer. Cell Res..

[2] Le Naour A., Rossary A., Vasson M.P. (2020). EO771, is it a well-characterized cell line for mouse mammary cancer model? Limit and uncertainty. Cancer Med..

[3] Gong Y., Ji P., Yang Y.S., Xie S., Yu T.J., Xiao Y., Jin M.L., Ma D., Guo L.W., Pei Y.C. (2021). Metabolic-Pathway-Based Subtyping of Triple-Negative Breast Cancer Reveals Potential Therapeutic Targets. Cell Metab..

[4] Yu X., Jia S., Yu S., Chen Y., Zhang C., Chen H., Dai Y. (2023). Recent advances in melittin-based nanoparticles for antitumor treatment: From mechanisms to targeted delivery strategies. J. Nanobiotechnol..

[5] Laurindo L.F., de Lima E.P., Laurindo L.F., Rodrigues V.D., Chagas E.F.B., de Alvares Goulart R., Araujo A.C., Guiguer E.L., Pomini K.T., Rici R.E.G. (2024). The therapeutic potential of bee venom-derived Apamin and Melittin conjugates in cancer treatment: A systematic review. Pharmacol. Res..

[6] de Oliveira A.M.B., Amparo T.R., Sant’Anna M.B.M., Picolo G., Almeida T.C. (2026). Bee Venom and Cancer: A Mini-review Focusing on Melittin Antitumoral Effects. Curr. Drug Targets.

[7] Duan X., Zou H., Yang J., Liu S., Xu T., Ding J. (2024). Melittin-incorporated nanomedicines for enhanced cancer immunotherapy. J. Control. Release.

[8] Majchrzak A., Lewandowski F., Hrynkiewicz R., Poniewierska-Baran A., Bebnowska D., Niedzwiedzka-Rystwej P. (2025). Granzyme B and melittin in cancer immunotherapy: Molecular mechanisms and therapeutic perspectives in head and neck cancers. Front. Immunol..

[9] Zhang T., Bai L., You R., Yang M., Chen Q., Cheng Y., Qian Z., Wang Y., Liu Y. (2024). Homologous-targeting biomimetic nanoparticles co-loaded with melittin and a photosensitizer for the combination therapy of triple negative breast cancer. J. Mater. Chem. B.

[10] Wang D., He J., Dong J., Wu S., Liu S., Zhu H., Xu T. (2021). UM-6 induces autophagy and apoptosis via the Hippo-YAP signaling pathway in cervical cancer. Cancer Lett..

[11] Ye R., Zheng Y., Chen Y., Wei X., Shi S., Chen Y., Zhu W., Wang A., Yang L., Xu Y. (2021). Stable Loading and Delivery of Melittin with Lipid-Coated Polymeric Nanoparticles for Effective Tumor Therapy with Negligible Systemic Toxicity. ACS Appl. Mater. Interfaces.

[12] Wu R., Li N., Huang W., Yang Y., Zang R., Song H., Shi J., Zhu S., Liu Q. (2025). Melittin suppresses ovarian cancer growth by regulating SREBP1-mediated lipid metabolism. Phytomedicine.

[13] Wang Y., Yuan T., He L., Huang J., Wilfred N., Yang W., Jin M., Huang G., Lu C. (2024). Melittin treatment suppressed malignant NSCLC progression through enhancing CTSB-mediated hyperautophagy. Biomed. Pharmacother..

[14] Kashyap S., Patidar V., Kumar A., Sahu P., Dhiman M., Garg P., Jain A., Munshi A. (2026). Proteomic signatures in triple-negative breast cancer. J. Proteom..

[15] Vasiyani H., Mane M., Rana K., Shinde A., Roy M., Singh J., Gohel D., Currim F., Srivastava R., Singh R. (2022). DNA damage induces STING mediated IL-6-STAT3 survival pathway in triple-negative breast cancer cells and decreased survival of breast cancer patients. Apoptosis.

[16] Motiei M., Aboutalebi F., Forouzanfar M., Dormiani K., Nasr-Esfahani M.H., Mirahmadi-Zare S.Z. (2021). Smart co-delivery of miR-34a and cytotoxic peptides (LTX-315 and melittin) by chitosan based polyelectrolyte nanocarriers for specific cancer cell death induction. Mater. Sci. Eng. C Mater. Biol. Appl..

[17] Lv S., Sylvestre M., Song K., Pun S.H. (2021). Development of D-melittin polymeric nanoparticles for anti-cancer treatment. Biomaterials.

[18] Guo Y., Zhang X., Wang S.Z., Feng H.H., Wu S.Y., Wu F.G. (2023). Metal-Phenolic Network-Facilitated “Foe-to-Friend” Conversion of Melittin for Cancer Immunotherapy with Boosted Abscopal Effect. Research.

[19] Bianchini G., De Angelis C., Licata L., Gianni L. (2022). Treatment landscape of triple-negative breast cancer—Expanded options, evolving needs. Nat. Rev. Clin. Oncol..

[20] Zagami P., Carey L.A. (2022). Triple negative breast cancer: Pitfalls and progress. npj Breast Cancer.

[21] Duffy C., Sorolla A., Wang E., Golden E., Woodward E., Davern K., Ho D., Johnstone E., Pfleger K., Redfern A. (2020). Honeybee venom and melittin suppress growth factor receptor activation in HER2-enriched and triple-negative breast cancer. npj Precis. Oncol..

[22] Jimenez-Rodriguez T.W., Ahmetaj L., Alfaya Arias T., Boni E., Bugadze L., Chauhan J., De Las Vecillas L., Ferastraoaru D., Izquierdo E., Karagiannis S.N. (2026). Melittin in AllergoOncology: From Honey Bee Venom to Anti-Cancer Therapeutic Innovation-An EAACI Position Paper. Allergy.

[23] Krug K., Jaehnig E.J., Satpathy S., Blumenberg L., Karpova A., Anurag M., Miles G., Mertins P., Geffen Y., Tang L.C. (2020). Proteogenomic Landscape of Breast Cancer Tumorigenesis and Targeted Therapy. Cell.

[24] Yamaguchi H., Condeelis J. (2007). Regulation of the actin cytoskeleton in cancer cell migration and invasion. Biochim. Biophys. Acta.

[25] He Y., Sun M.M., Zhang G.G., Yang J., Chen K.S., Xu W.W., Li B. (2021). Targeting PI3K/Akt signal transduction for cancer therapy. Signal Transduct. Target. Ther..

[26] Mandal R., Barron J.C., Kostova I., Becker S., Strebhardt K. (2020). Caspase-8: The double-edged sword. Biochim. Biophys. Acta Rev. Cancer.

[27] Kenis H., van Genderen H., Bennaghmouch A., Rinia H.A., Frederik P., Narula J., Hofstra L., Reutelingsperger C.P. (2004). Cell surface-expressed phosphatidylserine and annexin A5 open a novel portal of cell entry. J. Biol. Chem..

[28] Kang T.H., Park J.H., Yang A., Park H.J., Lee S.E., Kim Y.S., Jang G.Y., Farmer E., Lam B., Park Y.M. (2020). Annexin A5 as an immune checkpoint inhibitor and tumor-homing molecule for cancer treatment. Nat. Commun..

[29] Szklarczyk D., Nastou K., Koutrouli M., Kirsch R., Mehryary F., Hachilif R., Hu D., Peluso M.E., Huang Q., Fang T. (2025). The STRING database in 2025: Protein networks with directionality of regulation. Nucleic Acids Res..

[30] Shannon P., Markiel A., Ozier O., Baliga N.S., Wang J.T., Ramage D., Amin N., Schwikowski B., Ideker T. (2003). Cytoscape: A software environment for integrated models of biomolecular interaction networks. Genome Res..

[31] Mammoto A., Huang S., Moore K., Ingber D.E. (2004). Role of RhoA, mDia, and ROCK in cell adhesion, migration and mechanotransduction. J. Biol. Chem..

[32] Davis R.J. (2000). Signal transduction by the JNK group of MAP kinases. Cell.

[33] O’Shea J.J., Schwartz D.M., Villarino A.V., Gadina M., McInnes I.B., Laurence A. (2015). The JAK-STAT pathway: Impact on human disease and therapeutic intervention. Annu. Rev. Med..

[34] Babst M. (2011). MVB vesicle formation: ESCRT-dependent, ESCRT-independent and everything in between. Curr. Opin. Cell Biol..

[35] Komander D., Rape M. (2012). The ubiquitin code. Annu. Rev. Biochem..

